# Associations between school-based peer networks and smoking according to socioeconomic status and tobacco control context: protocol for a mixed method systematic review

**DOI:** 10.1186/s13643-019-1225-z

**Published:** 2019-12-06

**Authors:** H. J. Littlecott, J. Hawkins, M. Mann, G. J. Melendez-Torres, F. Dobbie, G. Moore

**Affiliations:** 1 Centre for the Development and Evaluation of Complex Interventions for Public Health Improvement (DECIPHer), 1-3 Museum Place, Cardiff, CF10 3BD UK; 2 Specialist Unit for Review Evidence (SURE), Cardiff University, Level 5, Neuadd Meirionnydd, Cardiff, CF10 3AT UK; 3 Peninsula Technology Assessment Group, College of Medicine and Health, University of Exeter, Exeter, UK; 4 Usher Institute, University of Edinburgh, Doorway 1, Old Medical School, Teviot Place, Edinburgh, EH8 9AG UK

**Keywords:** Social network, Peer effects, Friendship, Social influence, Smoking, Mixed method

## Abstract

**Background:**

Smoking remains a major public health concern. School-based social networks influence uptake of smoking among peers. During the past two decades, the UK macro-systemic context within which schools are nested and interact with has changed, with anti-smoking norms having become set at a more macro-systemic level. Whilst the overall prevalence of smoking in the UK has decreased, inequality has prevailed. It is plausible that the influence of school-based social networks on smoking uptake may vary according to socioeconomic status. Therefore, this study aims to understand how social influence on smoking among adolescents has changed in line with variance within and between contexts according to time and geography.

**Methods:**

The following databases will be searched: Medline, PsycINFO, Embase, Applied Social Sciences Index and Abstracts (ASSIA), British Education Index, Sociological abstracts, Cumulative Index to Nursing and Allied Health Literature (CINAHL), Education Resources Information Center (ERIC) and Scopus. Additional searches will include reference checking of key papers, citation tracking, word of mouth and grey literature searches. The search strategies will incorporate terms relating to smoking, adolescents, schools, peers, network analysis and qualitative research. Titles and abstracts and full texts will be independently screened and assessed for quality by at least two researchers. Included studies will be assessed for quality, and data will be extracted for synthesis, including participant characteristics, setting and tobacco control context, study design and methods, analysis and results and conclusions. Quantitative findings will be narratively synthesised, whilst a lines of argument synthesis combined with refutational analysis will be employed to synthesise qualitative data. Both sets of findings will be charted on a timeline to add context to network findings and obtain an enhanced understanding of changes over time.

**Discussion:**

This protocol is for a mixed methods synthesis of both social network findings, to investigate social structures and qualitative studies, to elicit contextual information. The review will synthesise changes in the context of social influence on adolescent smoking over time and geographically. As context is increasingly recognised as a key source of complexity, this enhanced understanding will help to inform future interventions targeting smoking through social influence. This will help to enhance their relevance to context, subsequent effectiveness and targeting of inequalities.

**Systematic review registration:**

PROSPERO CRD42019137358

## Background

Whilst youth smoking is at an all-time low, smoking uptake remains a major public health concern; recent data indicate for example that nearly 1 in 10 young people in Wales, United Kingdom (UK), leave school as smokers [1]. Many adult smokers began smoking in adolescence, with nearly 40% becoming addicted before the age of 16 [2]. Moreover, the risk of smoking-related disease increases as the years of smoking and the number of cigarettes per day increase, and these factors are more prevalent among smokers who have initiated at a young age [3]. Therefore, it remains imperative to target adolescent smoking in order to improve disease outcomes in later life.

Smoking is often described as a ‘socially contagious’ behaviour, and key risk factors for smoking relate to the smoking behaviours, attitudes and norms of important others, such as parents [4] and peers [5]. Whilst, as open complex systems [6, 7], schools are influenced by macro-systems, such as local authorities, and contain micro-systems, such as year groups and classes, schools can be conceptualised as bounded micro-systems for the purpose of analysing social networks. Social networks within the school as a whole, or within sub-systems, may be supportive or opposed to smoking and will influence the uptake of smoking among peers. Valente et al. [5] employed social network analysis to show that ‘popular’ children who had more leverage over school norms were themselves more likely to be smokers or to view smoking as acceptable. To date, both primary research and systematic reviews have demonstrated the utility of social network analysis in investigating the link between peer influence and tobacco smoking as well as understanding complex systems [8]. Abel and colleagues [9] advocate for visual display of networks, cluster analysis and qualitative data to triangulate and enhance understanding of the context surrounding social interactions.

Intervention models such as A Stop Smoking In Schools Trial (ASSIST) have focused on harnessing peer influence within schools to prevent smoking [10]. ASSIST was shown to be effective at the time, with subgroup analyses evidencing a higher level of effectiveness in schools located in the South Wales Valleys, where schools had a lower socioeconomic composition, smoking rates were higher and there was a high social network density (actual number of ties in relation to potential ties) [10]. During the past two decades, the UK macro-systemic context within which schools are nested and interact with has changed, with anti-smoking norms having become set at a more macro-systemic level. The Context and Implementation of Complex Interventions (CICI) framework [11] states that three concepts interact to affect intervention outcomes: context, implementation and the setting, where context comprises geographical, epidemiological, socio-cultural, socio-economic, ethical, legal and political factors. Changes have occurred across a range of these domains which may impact how interventions like ASSIST work. For example, in the UK, legislation has been introduced to restrict marketing, such as advertising, sports and events sponsorship and increase taxation in line with inflation [12]. Smoking was banned in public places in the UK between 2006 and 2007, with the acceptance of this legislation in many international contexts reflecting changing norms, whilst the legislation in turn serves to further accelerate denormalisation of smoking, with implications for its subsequent prevalence among children and adults [13]. This was followed by legislation on the display of tobacco products at point of sale through the Health Act in 2009 [14], smoking in cars carrying children in 2014 [15] and the introduction of plain packaging for tobacco products in 2016 [16].

Lorant et al. [17] investigated the role of school-based social networks, focusing on how these may relate to inequalities in smoking among adolescents. They found that less affluent adolescents had a higher exposure to friends who smoked and were more likely to smoke themselves. Moreover, whilst the prevalence of smoking in the UK has decreased, inequality has prevailed [13, 18, 19], with recent studies indicating that pupils from poorer families, and poorer schools, remain more likely to initiate smoking [20, 21]. This growing inequality combined with lower overall prevalence demonstrates a major change in the epidemiological context of smoking. This may mean that interventions based on peer influence, such as ASSIST [10], may need to be adapted in order to work effectively across different school contexts, which vary by socioeconomic composition [22]. Current understandings of social influence within schools have largely framed social network processes as if they are universal across schools, neglecting to incorporate contextual influences [22].

Social diffusion models work through influencing group norms. It is plausible that the potential influence of school-based social networks on young people’s smoking uptake may vary according to socioeconomic status (SES). For example, in more affluent school contexts, smoking is more likely to be a highly stigmatised behaviour, restricted to more ‘deviance prone’ individuals [23]. Thus, it may be increasingly difficult to reach and influence smokers, who could be isolated from school norms [24, 25]. Where smoking remains more normalised, often in more deprived schools, using popular and influential students to influence school social networks may still be an effective mechanism to decrease smoking prevalence [25].

In support of this, simulation models have hypothetically estimated that differences in initial smoking prevalence within a school context can lead to highly differential effects on adolescent smoking, arising from the same intervention [26]. This may challenge the assumption of earlier studies of smoking and social networks, which assumed a universal tendency for the ‘popular’ students to be smokers [5], particularly in light of anti-smoking legislation, which has accelerated denormalisation and contributed to rapidly changing smoking contexts over the past decade. Thus, the dominant role of peers may have shifted over time from causing smoking to sustaining abstinence through social disapproval. This change in context may indicate that population-level impacts of peer norm smoking interventions are likely to be weakened and to elicit differential effects across contexts [13, 27]. Thus, whilst peer norm interventions, such as ASSIST, were cost-effective when smoking was more widespread, it is possible that implementing such interventions within less affluent schools, where social norms continue to support smoking uptake, may be more cost-effective in the current context. Despite this, there is still a paucity of interventions which measure, or are sensitive to, differential processes and outcomes across socioeconomic contexts [13, 28].

In summary, there is a need to revisit our understandings of social influence on smoking internationally to inform subsequent exploration of this relationship and context-specific intervention design within the UK, where smoking has become increasingly denormalised. Within this review, the main focus will be upon the geographical, epidemiological, socio-cultural, socioeconomic and legal context [11]. This protocol outlines a mixed method synthesis of both social network findings, to investigate social structures, and qualitative studies of factors relating to smoking uptake, to elicit contextual information on social influence on smoking among adolescents. It is imperative that we understand the extent to which these separate sets of literature show whether and how social influence on adolescent smoking has changed over time, and the extent to which they vary by SES and tobacco control context. This will help to inform future peer-based interventions, which may be adapted according to context.

## Review aim and questions

The review aims to synthesise and combine quantitative and qualitative data to understand how social influence on smoking among adolescents has changed in line with the variance within and between contexts according to time and geography.

1. What are the perceptions of, and associations between, school-based social networks and smoking among adolescents?
  - To what extent, why and how do these vary by SES?
  - To what extent, why and how do these vary between countries, and over time, according to the proximity of the introduction of a smoking ban at the time of data collection?

## Methods

The present review protocol is being reported in accordance with the reporting guidance the Preferred Reporting Items for Systematic Review and Meta-Analysis Protocols (PRISMA-P) statement [29] (see PRISMA-P checklist in Additional file 1). This review was registered with the International Prospective Register of Systematic Reviews (PROSPERO) (registration number: CRD42019137358).

### Information sources and search strategy

The following databases will be searched for abstracts, full texts and conference proceedings: Medline, PsychINFO, Embase, Applied Social Sciences Index and Abstracts (ASSIA), British Education Index, Sociological abstracts, Cumulative Index to Nursing and Allied Health Literature (CINAHL), Education Resources Information Center (ERIC) and Scopus. The secondary source of potentially relevant material will be a search of the grey or difficult to locate literature, including two dissertation databases: the ProQuest Dissertations and Theses database and the Network Digital Library of Theses and Dissertations. We will perform hand-searching of the reference lists of included studies, relevant reviews or other relevant documents.

The two searches of quantitative and qualitative literature will employ a strategy to include terms relating to smoking, schools, adolescents and peers. In addition, the search strategy for quantitative literature will include terms related to social networks, and the search strategy for qualitative literature will include a qualitative search filter adapted from a previous qualitative systematic review [30]. The draft Medline search strategies are included in Additional file 2.

### Eligibility criteria

Both the searches for quantitative and qualitative data will include studies which meet the following criteria. Papers published from 1997 onwards, using data from 1997 onwards with abstracts written in English will be considered. This is the year that adolescent smoking peaked in the USA [31]. This will allow for the review to capture changes over time from a period of normalisation to the contemporary situation in which smoking is now highly denormalised in many Western societies.

The population will be school students (age 11–18), school staff, parents or other education professionals. Included studies will be focused on whole population or students of a low socioeconomic status. Studies focused on special populations will be excluded. No geographical limits will be set, but comparisons will be made within the analyses between countries that have and have not banned smoking in public places.

### Quantitative eligibility criteria

The quantitative search criteria have been guided by the Population Exposure Comparator Outcome (PECO) framework (see Table 1) [32]. Quantitative studies will have the population as stated above and will employ a social network design. Studies conducting network analysis will employ either a cross-sectional or longitudinal cohort design to study participants’ exposure to network characteristics, such as centrality (to measure popularity and gatekeeping) and density (actual number of ties in relation to potential ties) will be considered. The primary outcome of these studies of interest will be the link between network characteristics and smoking behaviour and attitudes. Secondary outcomes will include network characteristics and smoking prevalence. Comparators will not be applicable within this review.

**Table 1:** Eligibility criteria using the PECO format

Criterion	Definition
(P) Population	• Secondary school students (age 11–18) • Focused on whole population or students of a low socioeconomic status • Papers published from 1997 onwards, using data from 1997 onwards • Papers with abstracts written in English
(E) Exposure	• Network characteristics (number of ties, network structure, betweenness and degree centrality, density) • Any study conducting cross-sectional or longitudinal network analysis
(C) Comparator	• Not applicable
(O) Outcome	• Link between network characteristics and smoking behaviour and attitudes • Network characteristics • Smoking prevalence

### Qualitative eligibility criteria

The qualitative search criteria have been guided by the Sample, Phenomenon of Interest, Design, Evaluation, Research type (SPIDER) framework (see Table 2) [33]. The sample for these studies has been described above. Membership of friendship groups and smoking behaviour of peers will be the phenomenon of interest for qualitative studies. Qualitative study designs or mixed methods studies with a qualitative element will be considered. The main evaluation outcome of interest will be participants’ perceptions of friendship and peers and how they may, or may not, influence smoking behaviour and attitudes.

**Table 2:** Eligibility criteria using SPIDER format

Criterion	Definition
(S) Sample	• Secondary school students (age 11–18), school staff, parents or other education professionals • Focused on whole population or students of a low socioeconomic status • Papers published from 1997 onwards, using data from 1997 onwards • Papers with abstracts written in English
(PI) Phenomenon of interest	• Friendship groups and peers
(D) Study design	• Qualitative • Mixed method with a qualitative element
(E) Evaluation outcomes	• Perceptions of the link between friendship, peers and smoking • Perceptions of smoking
(R) Research type	• Qualitative

## Screening, selection and data extraction

The studies identified will be imported into Endnote software for screening. Titles and abstracts will be screened independently by two researchers employing the inclusion and exclusion criteria. This process will then be repeated with the full texts obtained for each study whose title and abstract met the inclusion criteria. Discrepancies will be resolved by a third reviewer. The following categories of information will then be extracted from full texts by each of the two researchers using a data extraction form. Date of data collection will be noted. Participant characteristics will be extracted, such as number of schools and individuals and their age, gender and socioeconomic status. Details of the setting and tobacco control context, such as the country and the timing and extent of any smoking ban that has been introduced, will be extracted and/or sourced from elsewhere. Details of the study design and methods will be extracted, including the overall study design as well as what type of ties were captured and how they were collected. Analysis procedures, such as the software used and statistical analysis or qualitative analysis techniques employed and whether any sub-group analysis was undertaken will be sought. Finally, the results and conclusions, such as network characteristics and their association with smoking or participants’ perceptions of how friendship and peers may or may not influence smoking behaviour and attitudes will be recorded. Extraction forms will be developed separately for quantitative and qualitative data. They will then be piloted and edited accordingly, to ensure that the forms are user-friendly and that they capture the most relevant information.

## Risk of bias (quality) assessment

All included studies will be independently appraised for quality by two researchers. Critical appraisal will be undertaken using the Specialist Unit for Review Evidence (SURE)’s Cross-Sectional Studies and Cohort Studies Checklists [34, 35] and the Evidence for Policy and Practice Information and Co-ordinating Centre (EPPI-centre) tool for the appraisal of qualitative studies [36].

SURE’s Cross-Sectional Studies and Cohort Studies Checklists include 12–13 items on study design, reporting and awareness of limitations. The EPPI-Centre tool for the appraisal of qualitative studies includes 8 items on rigour, findings and whether the perspectives of children are privileged. Studies are also rated low, medium or high according to the weight one would assign to for the trustworthiness of findings and the usefulness of each study for the review.

## Synthesis

### Synthesis of quantitative data

Due to the nature of social network data, we anticipate that a meta-analysis will not be possible. Instead, studies will be grouped according to both a priori defined groupings and those that emerge inductively as the data are analysed. Studies will be charted against a timeline and narratively synthesized to identify changes over time.

### Synthesis of qualitative data

A key paper integration will be undertaken with publications ordered thematically and charted against a timeline [37]. A lines-of-argument synthesis to build a picture of the whole through translating studies into each other [38] will then be combined with refutational analysis [38] to identify and analyse incommensurate areas between studies and how findings have changed over time. A meta-narrative lens will be applied to obtain an understanding of how different paradigms may have influenced this field. Meta-narrative reviews focus on an unfolding storyline of how fields have changed over time, thus providing a methodology through which to understand true changes in the social influence of smoking over time, in line with the smoking ban, and the extent to which methodological advances and paradigm shifts may have had a role in these advances in understanding and changing results [39].

### Integration

Quantitative and qualitative findings will be integrated using a convergent qualitative synthesis [40] in order to add context to network findings and obtain an enhanced understanding of changes over time. Firstly, quantitative network data will be qualitised, before combining the key themes from both quantitative and qualitative studies by visually charting them against a timeline to combine both chronological perspectives to understand how and why social influence on adolescent smoking has changed over time.

## Discussion

To date, both primary research and systematic reviews have demonstrated the social influence of smoking among adolescents [41]. However, social network and qualitative data have yet to be combined to achieve a greater understanding of the context within which this social influence takes place. The rapidly changing smoking contexts of the past decade [18, 42] have implications for related interventions, as context is increasingly recognised as a key source of complexity [43]. The current review of the literature will aim to achieve this enhanced understanding across geographical and temporal contexts through employing mixed methods. This will help to inform future interventions targeting smoking through social influence to enhance their relevance to context, subsequent effectiveness and targeting of inequalities.

The main strength of this review is the synthesis of mixed methods using novel analysis techniques, whilst the main limitation is the need to limit inclusion criteria in terms of date of data collection. However, the year 1997 was chosen due to this being the year that smoking peaked in the USA [31] and this was combined with no geographical limits. This will allow a meaningful analysis to be conducted into change across temporal and geographical contexts. Further potential limitations include difficulties comparing studies which vary in terms of both their methodology and data collected; thus, we do not anticipate being able to perform a meta-analysis. Moreover, the conclusions drawn from the synthesis results will be dependent upon the quality of the literature available, and whilst a comprehensive search is planned, it is possible that some relevant studies may still be missed. Any deviations from the protocol will be agreed by investigators and logged for reporting with the completed systematic review.

## Supplementary information

**Additional file 1.** PRISMA-P checklist.

**Additional file 2: Table S1.** MEDLINE search strategy for quantitative data. **Table S2.** MEDLINE search strategy for qualitative data.

## Data Availability

Data to support the findings of this systematic review will be made available through the Systematic Review Data Repository (SRDR) after the completion of the review.

## Abbreviations

ASSIA: Applied Social Sciences Index and Abstracts
ASSIST: A Stop Smoking In Schools Trial
CICI Framework: Context and Implementation of Complex Interventions Framework
CINAHL: Cumulative Index to Nursing and Allied Health Literature
EPPI-Centre: Evidence for Policy and Practice Information and Co-ordinating Centre
ERIC: Education Resources Information Center
PECO: Population Exposure Comparator Outcome
PRISMA-P: Preferred Reporting Items for Systematic Review and Meta-Analysis Protocols
SES: Socioeconomic status
SPIDER: Sample, Phenomenon of Interest, Design, Evaluation, Research type
SURE: Specialist Unit for Review Evidence
UK: United Kingdom
