# Supplementary material for: Associations between school-based peer networks and smoking according to socioeconomic status and tobacco control context: protocol for a mixed method systematic review
Source: Syst Rev. 2019 Dec 6;8:313. doi: 10.1186/s13643-019-1225-z (PMC6896310; doi:10.1186/s13643-019-1225-z)
Supplement: Supplementary file 2 — Additional file 2: Table S1. MEDLINE search strategy for quantitative data. Table S2. MEDLINE search strategy for qualitative data. [file 13643_2019_1225_MOESM2_ESM.docx]

Table S1. MEDLINE Search strategy for quantitative data.

| 1 smoking/  2 exp Cigarette Smoking/  3 smoking.ti,ab.  4 ((smok* or tobacco or cigarette* or nicotine or substance) adj3 (addict* or use* or usage or  using or intake or consum*)).ti,ab.  5 ((smok* or tobacco or cigarette* or nicotine) adj3 (prevalence or behavio?r)).ti,ab.  6 or/1-5  7 exp schools/  8 secondary schools/  9 ((middle or junior high or high or secondary or secondary) adj3 (school or educat*)).tw.  10 or/7-9  11 (student* or pupil* or young adult* or young person or juvenile* or young people).tw.  12 (adolesc* or teen* or minor* or boy* or girl* or youth*).tw.  13 exp Adolescent/ or exp Minors/ or exp Young Adult/  14 or/11-13  15 social networking/  16 ("social network*" adj2 (analys* or method* or approach or process* or influence*)).ti,ab.  17 (central* adj (closeness or betweenness or degree)).ti,ab.  18 (social network* or network or density or isolate* or clique* or liaison* or gatekeeper*).mp.  19 *"Social Behavior"/  20 Peer Group/  21 Friends/ (14998)  22 Interpersonal Relations/  23 Social Behavio?r/  24 (school-based network or network structure).tw.  25 ((peer* or friend*) adj3 (pressure or influence or selection or norm* or role or network*)).tw.  26 social capital/ or social isolation/ or social norms/  27 peer influence/  28 peers/ or peer relations/ or friendship/ or social groups/  29 or/15-28  30 6 and 10 and 14 and 29  31 (network* adj5 smoking).tw.  32 30 or 31  33 limit 32 to yr="1997 -Current" |
| --- |

Table S2 MEDLINE Search strategy for qualitative data.

| 1 smoking/  2 exp cigarette smoking/  3 smoking.ti,ab.  4 ((smok* or tobacco or cigarette* or nicotine) adj3 (addict* or use* or usage or using or intake or  consum*)).ti,ab.  5 ((smok* or tobacco or cigarette* or nicotine) adj3 (prevalence or behavio?r)).ti,ab.  6 or/1-5  7 (student* or pupil* or young adult* or young person or juvenile* or young child*).tw.  8 (adolesc* or teen* or minor* or boy* or girl* or youth* or young).tw.  9 exp Adolescent/ or exp Minors/ or exp Young Adult/  10 or/7-9  11 Social behavior/  12 peer group/  13 friends/  14 Interpersonal relations/  15 social behavio?r.tw  16 social capital/ or social isolation/ or social norms/  17 peer influence/  18 peers/ or peer relations/ or friendship/ or social groups/  19 ((peer* or friend*) adj3 (pressure or influence or selection or norm* or role)).mp.  20 or/11-19  21 exp Qualitative research/  22 qualitative research.mp.  23 Interview/ or interview.mp.  24 ((interview* or focus group*) adj3 qualitative).mp.  25 focus groups/ or focus group.mp.  26 (qualitative adj3 (study or research or method* or analysis or cod* or them* or question*1 or data)).ti,ab.  27 (thematic analysis or ethnological research or ethnograph*).ti,ab.  28 (theme*1 adj2 (qualitative or analysis or coding or codes or grouping or identif*)).ti,ab.  29 (grounded adj (theor* or study or studies or research or analys?s)).mp.  30 (data adj1 saturat*).ti,ab.  31 (field adj (study or studies or research)).ti,ab.  32 (perception* or thought* or knowledge or perspective* or attitude* or opinion* or idea* or "point of view" or  belie* or viewpoint* or understand* or representation* or views or experience*).mp.  33 or/21-32  34 6 and 10 and 20 and 33  35 limit 88 to yr="1997-current" |
| --- |
